# Outcome of neonatal hypoxemic respiratory failure: a livebirth population-based retrospective survey

**DOI:** 10.1186/s12887-022-03603-9

**Published:** 2022-09-17

**Authors:** Sufang Ding, Yaling Xu, Hui Wang, Hongni Yue, Zhaojun Pan, Bo Sun, on behalf of the Huai’an Perinatal-Neonatal Study Group, Guofang Zheng, Xiaoqin Zhu, Weijie Ding, Xiaoqiong Li, Tingting Qi, Muling Zhang, Zhaofang Tian, Honghua Guan, Juan Yang, Yongjian Wu, Tao Xu, Chunhong Tang, Maotian Dong, Chunhua Zhang, Chunqin Dong, Sumei Zhou, Yani Lei, Shouzhong Li, Keyan Zhu, Xia Zhao, Yaodong Yin, Haijun Wang, Bi Xue, Zhaoxia Wang, Shucheng Wang, Hong Liu, Zhou Xu, Chuntao Yuan, Xihui Cao, Jianya Zhang, Bu Xu, Wenlong Lin, Cui Gao, Yongbo Heng, Lei Wang, Moqing Wang

**Affiliations:** 1 Department of Neonatology, Huai’an Maternal and Child Health Care Center, Huai’an, 223002 Jiangsu China; 2 The National Commission of Health Laboratory of Neonatal Diseases, National Children’s Medical Center, Children’s Hospital of Fudan University, Shanghai, China; 3 Unit of Population Health Information, Huai’an Maternal and Child Health Care Center, Huai’an, Jiangsu China; 4 Departments of Pediatrics and Neonatology, Children’s Hospital of Fudan University, Shanghai, 201102 China

**Keywords:** Epidemiology, Livebirth, Morbidity, Mortality, Neonate, Respiratory distress syndrome, Respiratory failure, sepsis, Surfactant

## Abstract

**Background:**

To explore the prevalence, outcome and perinatal risks of neonatal hypoxemic respiratory failure (NRF) in a survey of all livebirths from a regional network of perinatal-neonatal care during the transition period after 5-year universal health insurance implemented in China.

**Methods:**

Clinical data of all neonatal respiratory morbidities in Huai’an were retrospectively collected in the regional perinatal network database of all livebirths as vital statistics in 2015. NRF was defined as hypoxemia requiring continuous positive airway pressure (CPAP) and/or mechanical ventilation (MV) for at least 24 h. Mortality risks of antenatal and perinatal morbidities, major respiratory therapies and complications were analyzed by multivariable logistic regression model.

**Results:**

There were 788 NRF cases identified in 9.9% (7960) hospitalized, or 13.3‰ (59056) livebirths, in which 6.7% received intensive care and 93.0% critical care. The major underlying morbidities were respiratory distress syndrome (RDS, 36.4%) and pneumonia/sepsis (35.3%), treated mainly by CPAP, MV and surfactant. Significantly improved outcomes by surfactant in RDS were in patients with birthweight (BW) < 1500 g or gestational age (GA) < 32 weeks. The overall mortality rate in NRF was 18.4% whereas for those of BW < 1000 g and GA < 28 weeks, 70% and 54%, respectively. The multivariable regression analysis showed the highest odds for NRF death among meconium aspiration syndrome, congenital anomalies, BW < 1500 g and necrotizing enterocolitis, whereas born in level III hospitals, cesarean delivery, CPAP and MV were associated with markedly reduced death odds.

**Conclusions:**

The salient findings with associated risk estimates reflected efficiency of respiratory support as critical care in a prefectural regional network infrastructure for annual livebirths in 5.6 million inhabitants. It implicated the representativeness of contemporaneous perinatal-neonatal care standard at medium to medium-high level, in one/fourth of the population of China, aiming at saving more life of very critical and preterm infants for better survival.

**Supplementary Information:**

The online version contains supplementary material available at 10.1186/s12887-022-03603-9.

## Introduction

In the past decade, the development of maternal-infant healthcare and modern perinatal-neonatal medicine has been in transition with universal health insurance implemented in China [1]. Almost all counties (about 2800 in number) have been establishing maternity and child healthcare hospitals/centers (more than 3000 in total), each serving for inhabitants from < 1/4 to > 1 million [2, 3]. Five to 15 of these counties consist of a sub-provincial metropolitan city, or prefectural region, and with a population from 1 to 2 million to 10 million. In nation-wide more than 300 such cities, there are > 500 level III, leading perinatal-neonatal care centers covering these cities/regions [3, 4].

Huai’an is such a city/region with 5.6 million inhabitants in 2015. Its gross domestic production (GDP, 44 billion USD equivalent, 1 USD =6.2 CNY), GDP per capita (9000 USD), and average disposable income (3300 USD) ranked 65th–70th, as medium or medium-high in ascending order, in all regional cities of the country [4, 5]. Its annual births were 50,000–60,000 in 2010–2019 [5]. We conducted a series of surveys to estimate incidences and risks of major outcomes in perinatal-neonatal care through Huai’an perinatal information system and perinatal-neonatal network. We have reported the rates of birth, livebirth, stillbirth, preterm birth, perinatal and neonatal mortality, including deaths at delivery, in hospital, and due to prematurity in Huai’an as vital statistic data in 2010 and 2015 [6, 7]. The database generated from these prospective surveys enabled integration of maternal obstetric information and in-hospital neonatal management and outcome, relevant for studies of various purposes [6–12]. For neonatal intensive and critical care, its availability and affordability constituted a foundation in view of equity, efficiency and equipoise from regional healthcare policy, facility, along with socioeconomic and sociocultural development. In this regard, outcome of neonatal patients requiring critical care, especially those of hypoxemic respiratory failure (NRF), tends to be a good focus [1].

The standard of neonatal critical care involves resuscitation at birth, transportation, prompt admission, life sign monitoring, catheterization, and respiratory support with advanced ventilation strategies and medications [1, 13–15]. During the transition period of implementation of universal health insurance, these therapeutic modalities were adopted to a variable extent in different regions of China, depending on the local economy, health policy, facilities which included number and competency of staffs [1, 2, 13–16]. In our previous studies, a series of prospective or retrospective surveys of NRF were carried out through nation-wide, provincial or trans-provincial networks of neonatal intensive care units (NICUs) as collaborative study groups [16–22]. It enabled geographical and longitudinal assessment of efficiency in respiratory support, ventilation mode, surfactant, inhaled nitric oxide in neonates of different lung development and pathologies. These facilities were mostly in tertiary hospitals in metropolitans or municipalities and the results may not be extrapolated for the regional care standard, as information in those treated outside of the central NICUs was not accessible.

For this issue, on the basis of the whole regional birth population survey in Huai’an in 2015 [7], we retrospectively conducted the first comprehensive clinical epidemiological study in the regional livebirth and in-hospital population with the same definition and protocol of NRF as in previous studies [16–19, 21–23]. Our purpose was to delineate efficiency of respiratory support as mainstay of critical care for NRF in the whole regional network of NICUs associated with local socioeconomic status (SES) as background [6–12], which coincided with the initial implementation of universal health insurance and adaptation within ensuing years [1]. We postulated that the results should facilitate estimation of incidence of major respiratory diseases, case fatality rate (CFR) and mortality rate by livebirths. By characterizing regional perinatal-neonatal care specific development, it may be further translated into nation-wide burden of neonatal respiratory morbidity and mortality, and efficiency of neonatal critical care in the era of universal health insurance as integrated.

## Methods

### Study design and data collection

The study design was on the assumption that the 2015 birth datafile provided comprehensive information regarding fetal deaths/stillbirths, livebirths, preterm births, in-hospital patients as well as maternal and perinatal morbidities, and overall perinatal and neonatal survival data [7, 9–12]. NRF cases were identified from all the in-hospital datafile of regional level II and III hospitals, through the collaborative network and research program based on previous experience and protocols [16, 17, 19, 21–23]. The definition of NRF was clinically and blood gas confirmed hypoxemia requiring continuous positive airway pressure (CPAP) and/or intratracheal mechanical ventilation (MV) for at least 24 h, or withdrawal of treatment/deaths within 24 h [16, 17, 19, 21–23]. Retrospective data collection and analysis were conducted in 2018–2021, and for transferred cases, the data were regarded as single hospitalization. Huai’an Maternal and Child Health Care Center (HMCHCC), as the major referral center in the region, acted as coordination center for this study. The ethics committee of HMCHCC and Children’s Hospital of Fudan University approved the study protocols, and the informed consent from parents/guardians was waived as no specific intervention was applied [9–12].

Data of maternal and perinatal morbidities, as well as neonatal outcomes were included, with the diagnostic definitions consistent with previous studies [6–12]. The major underlying diseases of NRF were defined as the primary cause and categorized as reported [12, 16, 19, 21, 22]. Briefly, underlying diseases were categorized as respiratory distress syndrome (RDS), meconium aspiration syndrome (MAS), temporary respiratory insufficiency of the newborn (TRIN) [24], pneumonia/sepsis, congenital anomalies (CA) and intraventricular hemorrhage (IVH, grades III and IV), with definitions in Additional file 1. The subsequent pathophysiological conditions developed from those underlying diseases were considered as complications, such as hospital acquired pneumonia/sepsis (ventilation-associated pneumonia, catheter-related sepsis and other healthcare-related severe infection after 48 h of hospitalization), persistent pulmonary hypertension of the newborn (PPHN), pulmonary hemorrhage, bronchopulmonary dysplasia (BPD, moderate to severe), neurological impairment (hypoxic ischemic encephalopathy, bilirubin encephalopathy, periventricular leukomalacia, hypoglycemic brain damage), patent ductus arteriosus (of hemodynamical significance), necrotizing enterocolitis (NEC, Bell’s stage II and III), retinopathy of prematurity (ROP, stage 3 and above) [10, 12, 25]. Information of respiratory support (CPAP, MV), surfactant, postnatal steroids, length of ventilation (LOV) including both CPAP and MV, length of hospital stay (LOHS), costs and survival at discharge, was also included. Score for neonatal acute physiology perinatal extension (SNAPPE) II was used for assessment of initial illness severity, and retrospectively determined based on routine items and score scales in the first 12 h after admission to the NICU based on the original in-hospital clinical records [26]. According to the severity of diseases and the treatment strength, care level in NICU was classified into intensive or critical care (Additional file 1) [27].

For the description of the clinical characteristics of NRF patients by maturity status, the gestational age (GA) was stratified into: GA, < 28 weeks (extreme preterm), 28–31 weeks (very preterm), 32–33 weeks (moderate preterm), 34–36 weeks (late preterm), 37–38 weeks (early term), 39–41 weeks (full term, as reference), ≥42 weeks (post-term); and birthweight (BW): < 1000 g (extremely low BW), 1000–1499 g (very low BW), 1500–2499 g (low BW), 2500–3999 g (normal BW, as reference), ≥4000 g. GA < 37 weeks and BW < 2500 g were deemed as preterm and low BW (LBW), respectively.

### Statistical analysis

The statistical analysis was performed by software SPSS 23.0 (IBM, Chicago, IL). Continuous variables were presented as mean ± standard deviation (SD) or median [interquartile range (IQR)] or range. Categorical variables by GA or BW strata were presented as number and percentage (%) with comparisons by Chi-square test or Fisher’s exact test. Numbers needed to treat (NNT) was estimated by reciprocal (reverse ratio) of attributable risk difference of the survival rates for surfactant treatment. Mann-Whitney U test was applied for comparisons of continuous variables. Spearman rank correlation was used between SNAPPE-II in category of every 10-point increment and mortality rate, while Pearson correlation test was used for continuous variables. The prevalence or mortality rates and associated 95% confidence interval (CI) were estimated by a generalized linear Poisson regression model with empirical, robust standard errors but no explanatory variables [28, 29]. Factors associated with NRF deaths were identified through univariable logistic regression, and those with probability (*P*) < 0.10 were further analyzed by multivariable models with backward stepwise selection. The crude and adjusted odds ratio (OR) and 95%CI of the identified variables, as well as the Hosmer-Lemeshow test for goodness of fit, were estimated. *P* < 0.05 was considered statistically significant.

## Results

There were 788 NRF cases, with corresponding prevalence of 13.3 and 95%CI of 12.4–14.3 per 1000 livebirth (*n* = 59,056), accounting for 9.9% total hospitalizations (*n* = 7960). As Table 1 lists, compared to non-NRF, NRF patients had lower median GA and BW, and were more prone to higher prevalence of maternal morbidities, more cesarean delivery, multiple births, preterm births, LBW, male, Apgar score at 5 min < 7 and delivery resuscitation. They also tended to have higher perinatal comorbidities, such as asphyxia, RDS, congenital pneumonia, early onset sepsis and CA. Moreover, 70.7% of NRF were born at, and 98.5% were admitted to, level III hospitals, and 99.7% in NICU, all higher than those of non-NRF patients (*P* < 0.001). Admission within 7 postnatal days (PND) accounted for 96.2% of total NRF, while 88.5% on PND 1. NRF required longer hospital stay days and higher costs compared to non-NRF. The overall CFR of NRF was 18.4% (*n* = 145), with the corresponding livebirth population-based mortality rate of 2.5‰ (95%CI 2.1‰, 2.9‰). For deaths in NRF, contributing to 86.3% of total in-hospital deaths (*n* = 168), 65.6% were within 7 PND, 93.1% within 28 PND, and 55.9% as withhold/withdrawal from critical care.

**Table 1:** The perinatal-neonatal characteristics of neonatal respiratory failure from all hospitalized patients of regionally livebirth population

	NRF	Non-NRF	All hospitalized
Case numbers^a^	788 (9.9)	7172 (90.1)	7960 (100)
GA, weeks	34.6 [31.3, 38.3]	39.0 [37.3, 40.0]^***^	39.0 [37.0, 40.0]
BW, g	2215 [1600, 3000]	3300 [2900, 3630]^***^	3250 [2750, 3600]
**Maternal major morbidities**			
Hypertensive disorder of pregnancy	113 (14.3)	668 (9.3)^***^	781 (9.8)
Gestational diabetes mellitus	29 (3.7)	221 (3.1)	250 (3.1)
Anemia	156 (19.8)	1019 (14.2)^***^	1175 (14.8)
Premature rupture of membrane	220 (27.9)	1071 (14.9)^***^	1291 (16.2)
Antenatal steroids	302 (38.3)	635 (8.9)^***^	937 (11.8)
Placenta abnormality	95 (12.1)	252 (3.5)^***^	347 (4.4)
Umbilical cord abnormality	91 (11.5)	593 (8.3)^**^	684 (8.6)
Fetal distress	31 (3.9)	74 (1.0)^***^	105 (1.3)
**Delivery status**			
Cesarean delivery	494 (62.7)	3671 (51.2)^***^	4165 (52.3)
Amniotic fluid contamination	117 (14.8)	842 (11.7)^*^	959 (12.0)
Multiple births	111 (14.1)	438 (6.1)^***^	549 (6.9)
Male	476 (60.4)	4027 (56.1)^*^	4503 (56.6)
Preterm	528 (67.0)	1413 (19.7)^***^	1941 (24.4)
Low BW	453 (57.5)	905 (12.6)^***^	1358 (17.1)
SGA	44 (5.6)	370 (5.2)	414 (5.2)
Apgar 5 min < 7	96 (12.2)	65 (0.9)^***^	161 (2.0)
DR resuscitation	221 (28.0)	396 (5.5)^***^	617 (7.8)
Born in level III hospitals	557 (70.7)	2841 (39.6)^***^	3398 (42.7)
**Perinatal morbidities**			
Congenital anomalies	146 (18.5)	426 (5.9)^***^	572 (7.2)
Congenital heart diseases	100 (12.7)	202 (2.8)^***^	302 (3.8)
Congenital diaphragmatic hernia	5 (0.6)	1 (0.0)^***^	6 (0.1)
Asphyxia	397 (50.4)	797 (11.1)^***^	1194 (15.0)
Intraventricular hemorrhage (III-IV)	114 (14.5)	329 (4.6)^***^	443 (5.6)
Respiratory distress syndrome	287 (36.4)	26 (3.2)^***^	313 (3.9)
Meconium aspiration syndrome	13 (1.6)	10 (0.1)^***^	23 (0.3)
Congenital pneumonia	626 (79.4)	1983 (27.6)^***^	2609 (32.8)
Early-onset sepsis	223 (28.3)	595 (8.3)^***^	818 (10.3)
Patent ductus arteriosus	75 (9.5)	111 (1.5)^***^	186 (2.3)
**In-hospital care and outcome**			
Admitted to level III hospitals	776 (98.5)	4187 (58.4)^***^	4963 (62.3)
Admitted on PND 1	697 (88.5)	2749 (38.3)^***^	1115 (14.0)
Intensive care	53 (6.7)	1323 (18.4)^***^	3446 (43.3)
Critical care	733 (93.0)	382 (5.3)^***^	1376 (17.3)
Surfactant	219 (27.8)	20 (0.3)^***^	239 (3.0)
CPAP	637 (80.8)	276 (3.8)^***^	913 (11.5)
MV	348 (44.2)	28 (0.4)^***^	376 (4.7)
Postnatal steroids	124 (15.7)	135 (1.9)^***^	259 (3.3)
Antibiotics	750 (95.2)	5638 (78.6)^***^	6388 (80.3)
Length of hospital stay, d	17 [11, 27]	7 [4, 10]^***^	7 [5, 11]
Cost of stay, CNY, ×10^3^	19 [12, 34]	5 [3, 8]^***^	6 [4, 9]
In-hospital death	145 (18.4)	23 (0.3)^***^	168 (2.1)
0–6 (PND)	95 (12.1)	13 (0.2)^***^	108 (1.4)
7–27	40 (5.1)	10 (0.1)^***^	50 (0.6)
≥ 28	10 (1.3)	0^***^	10 (0.1)
Withdrawal of treatment	81 (10.3)	17 (0.2)^***^	98 (1.2)

All values are presented as median [interquartile range, IQR] or n (%) referring to total number in each column unless otherwise indicated

*Abbreviations*: *NRF* Neonatal respiratory failure, *GA* Gestational age, *BW* Birthweight, *SGA* Small for GA, *DR* Delivery room, *PND* Postnatal day(s), *CNY* Chinese Yuan

*P < 0.05, **P < 0.01 and ***P < 0.001 vs. NRF

^a^Numbers refer to the total hospitalized patients (7960) from 59,056 livebirths

### Morbidity and mortality

**Fig. 1:**
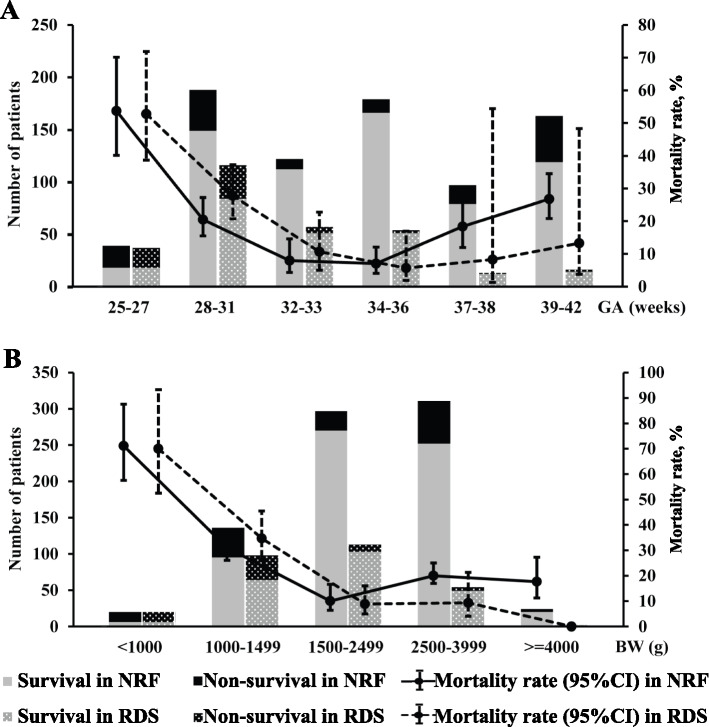
The incidence and mortality rate (95% CI) of neonatal respiratory failure and respiratory distress syndrome. **A** By GA strata. **B** By BW strata. NRF, neonatal respiratory failure; RDS, respiratory distress syndrome; GA, gestational age; BW, birthweight; CI, confidence interval

The main underlying morbidities of NRF were RDS (36.4%), pneumonia/sepsis (35.3%), TRIN (13.6%), CA (10.2%), MAS (1.6%) and IVH (2.9%), with corresponding CFR of each morbidity in NRF patients as 21.6%, 10.1%, 10.3%, 45.0%, 46.2% and 4.3%, respectively. In those extremely (BW < 1000 g or GA < 28 weeks) and very (BW < 1500 g or GA < 32 weeks) premature patients, RDS was diagnosed in approximately 100% and 70% with CFR of 50–70% and 40%, which contributed to 100% and 85% of NRF deaths, respectively (Table 2, Table S1, Fig. 1). The main complications were acquired pneumonia/sepsis (21.4%), neurological impairment (19.9%), while the occurrence of air leak, BPD, pulmonary hemorrhage, PPHN, NEC and ROP ranged from 4% to 10%.

**Table 2:** Primary diagnosis, intervention and outcome of NRF in BW strata

BW, g	< 1000	1000–1499	1500–2499	2500–3999	≥4000	Total
Livebirth	34	212	1602	49,106	8102	59,056
Death at delivery^a^	14 (41.2)	5 (2.4)	6 (0.4)	10 (0.0)	0	35 (0.1)
Hospitalization^a^	20 (58.8)	183 (86.3)	1155 (72.1)	5849 (11.9)	753 (9.3)	7960 (13.5)
NRF cases^a^	20 (58.8)	136 (64.2)	297 (18.5)	311 (0.6)	24 (0.3)	788 (1.3)
SNAPPE-II	28 [22, 43]	12 [5, 19]	5 [0, 16]	12 [5, 23]	10 [5, 21]	10 [5, 19]
Non-survivor	28 [20, 44]	16 [10, 30]	23 [14, 53]	44 [24, 63]	62 [14, 87]	30 [16, 53]
Survivor	31 [19, 47]	12 [5, 18]^***^	5 [0, 12]^***^	5 [5, 12]^***^	11 [5, 16]^**^	5 [0, 14]^***^
**Primary diagnosis**						
Respiratory distress syndrome	20 (100)	98 (72.1)	113 (38.0)	54 (17.4)	2 (8.3)	287 (36.4)
Survival^b^	6 (30.0)	64 (65.3)	103 (91.2)	49 (90.7)	2 (100)	225 (78.4)
Meconium aspiration syndrome	0	0	1 (0.3)	10 (3.2)	2 (8.3)	13 (1.6)
Survival^b^	0	0	1 (100)	4 (40.0)	2 (100)	7 (53.8)
Pneumonia/sepsis	0	28 (20.6)	99 (33.3)	139 (44.7)	12 (50.0)	278 (35.3)
Survival^b^	0	22 (78.6)	93 (93.9)	124 (89.2)	11 (91.7)	250 (89.9)
TRIN	0	8 (5.9)	46 (15.5)	49 (15.8)	4 (16.7)	107 (13.6)
Survival^b^	0	7 (87.5)	44 (95.7)	41 (83.7)	4 (100)	96 (89.7)
Congenital anomalies	0	1 (0.7)	29 (9.8)	46 (14.8)	4 (16.7)	80 (10.2)
Survival^b^	0	1 (100)	20 (69.0)	22 (47.8)	1 (25.0)	44 (55.0)
Intraventricular hemorrhage (III-IV)	0	1 (0.7)	9 (3.0)	13 (4.2)	0	23 (2.9)
Survival^b^	0	1 (100)	9 (100)	12 (92.3)	0	22 (95.7)
**Major complications**						
Acquired pneumonia/sepsis	13 (65.0)	54 (39.7)	55 (18.5)	41 (13.2)	6 (25.0)	169 (21.4)
Neurological impairment	5 (25.0)	33 (24.3)	57 (19.2)	56 (18.0)	6 (25.0)	157 (19.9)
Air leak	0	1 (0.7)	7 (2.4)	35 (11.3)	4 (16.7)	47 (6.0)
Bronchopulmonary dysplasia	6 (30.0)	46 (33.8)	15 (5.1)	5 (1.6)	0	72 (9.1)
Pulmonary hemorrhage	1 (5.0)	4 (2.9)	5 (1.7)	18 (5.8)	2 (8.3)	30 (3.8)
Persistent pulmonary hypertension	1 (5.0)	4 (2.9)	12 (4.0)	40 (12.9)	4 (16.7)	61 (7.7)
Patent ductus arteriosus	2 (10.0)	22 (16.2)	18 (6.1)	32 (10.3)	1 (4.2)	75 (9.5)
Necrotizing enterocolitis	7 (35.0)	22 (16.2)	14 (4.7)	1 (0.3)	2 (8.3)	46 (5.8)
Retinopathy of prematurity	4 (20.0)	22 (16.2)	6 (2.0)	2 (0.6)	1 (4.2)	35 (4.4)
**Respiratory interventions**						
Surfactant	16 (80.0)	82 (60.3)	85 (28.6)	36 (11.6)	0	219 (27.8)
CPAP	15 (75.0)	126 (92.6)	274 (92.3)	209 (67.2)	13 (54.2)	637 (80.8)
CPAP only	4 (20.0)	58 (42.6)	213 (71.7)	147 (47.3)	8 (33.3)	430 (54.6)
CPAP & MV	11 (55.0)	68 (50.0)	61 (20.5)	62 (19.9)	5 (20.8)	207 (26.3)
MV	15 (75.0)	75 (55.1)	82 (27.6)	160 (51.4)	16 (66.7)	348 (44.2)
MV initial	8 (40.0)	30 (22.1)	35 (11.8)	98 (31.5)	12 (50.0)	183 (23.2)
HFOV	7 (35.0)	15 (11.0)	16 (5.4)	44 (14.1)	3 (12.5)	85 (10.8)
Postnatal steroids	3 (15.0)	20 (14.7)	24 (8.1)	67 (21.5)	10 (41.7)	124 (15.7)
**Outcomes**						
Length of ventilation, hour	160 [47, 580]	124 [63, 255]	70 [41, 120]	55 [34, 166]	74 [41, 114]	70 [39, 126]
Non-survivor	84 [32, 206]	62 [27, 144]	36 [21, 120]	23 [6, 70]	92 [40, 114]	41 [13, 117]
Survivor	706 [155, 1439]^***^	160 [94, 310]^***^	71 [43, 121]	62 [40, 98]^***^	67 [41, 125]	72 [43, 134]^***^
Length of hospital stay, day	16 [4, 43]	38 [11, 54]	20 [14, 28]	13 [7, 17]	15 [10, 19]	17 [11, 27]
Non-survivor	7 [2, 22]	4 [1, 12]	3 [1, 16]	2 [1, 3]	6 [3, 9]	3 [1, 8]
Survivor	71 [38, 111]^***^	47 [35, 57]^***^	21 [15, 30]^***^	15 [11, 19]^***^	16 [12, 21]^***^	19 [14, 30]^***^
Costs of stay, CNY, ×10^3^	39 [15, 59]	45 [16, 64]	22 [15, 33]	15 [9.2, 21]	15 [12, 23]	19 [12, 34]
Non-survivor	18 [6.7, 45]	11 [3.6, 19]	5.9 [3.0, 14]	5.5 [2.3, 10]	12 [9.9, 20]	6.8 [3.3, 15]
Survivor	75 [44, 151]^***^	53 [42, 71]^***^	23 [16, 35]^***^	16 [12, 23]^***^	15 [12, 23]	21 [14, 36]^***^
Mortality	14 (70.0)	41 (30.1)	27 (9.1)	59 (19.0)	4 (16.7)	145 (18.4)
0–6 (PND)	7 (35.0)	25 (18.4)	16 (5.4)	45 (14.5)	2 (8.3)	95 (12.1)
7–27	4 (20.0)	11 (8.1)	11 (3.7)	12 (3.9)	2 (8.3)	40 (5.1)
≥ 28	3 (15.0)	5 (3.7)	0	2 (0.6)	0	10 (1.3)
Withdrawal of treatment	7 (35.0)	29 (21.3)	14 (4.7)	28 (9.0)	3 (3.7)	81 (10.3)

All values are presented as median [interquartile range, IQR] or n (%) referring to total NRF case number in each column

*Abbreviations*: *NRF* Neonatal respiratory failure, *BW* Birthweight, *SNAPPE-II* Score for neonatal acute physiology perinatal extension II, *TRIN* Transient respiratory insufficiency of the newborn, *CPAP* Continuous positive airway pressure, *MV* Mechanical ventilation, *HFOV* High frequency oscillatory ventilation, *CNY* Chinese Yuan

**P < 0.01, ***P < 0.001 vs. non-survivor

^a^Numbers refer to the total livebirths in each column

^b^Survival rate (%) refers to the specific case number listed above

**Fig. 2:**
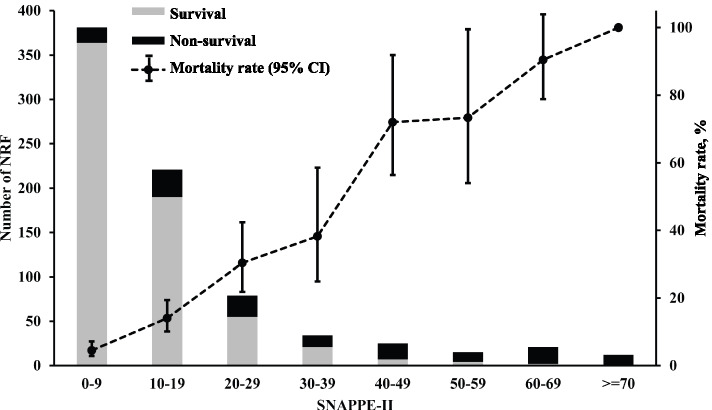
Distribution of SNAPPE-II in the incidence and mortality rate of neonatal respiratory failure. SNAPPE-II, score for neonatal acute physiology perinatal extension II; NRF, neonatal respiratory failure; CI, confidence interval

As Table 2 and Table S1 list, the overall mortality rates of NRF were 70% or 53.8% in those of BW < 1000 g or GA < 28 weeks, while 30.1% or 20.7% of BW 1000–1499 g or GA 28–31 weeks, respectively. The median SNAPPE-II scores were highest in those with BW < 1000 g or GA 25–27 weeks. As Fig. 2 shows, SNAPPE-II was strongly correlated with mortality rate of NRF (*r* = 0.895, *P* < 0.001).

### Respiratory support and surfactant use

The prevalence rates of CPAP, MV, surfactant and postnatal steroids use were 80.8%, 44.2%, 27.8% and 15.7%, respectively. In very premature patients, the utility rates of CPAP, MV and surfactant were nearly 90%, 50% and 60%, respectively (Table 2 and Table S1). The median values of LOV, LOHS, and costs of stay in the NRF survivors were 1.8, 6.3 and 3.1 times, respectively, of those deceased. The correlation coefficients of LOV and LOHS, LOV and costs, as well as LOHS and costs were 0.599, 0.623 and 0.754, respectively (all *P* < 0.001). The costs of rescuing a NRF patient were about 20,000 CNY, equivalent to a local adult’s annual disposable income. The financial burden was much higher in those of extremely or very preterm/LBW, about 80,000 CNY or 50,000 CNY, respectively. Table 3 depicts the overall efficiency of surfactant in RDS-related NRF patients. Surfactant improved survival rate mainly in the very and extremely preterm/LBW patients.

**Table 3:** Surfactant therapy and survival rate by GA or BW strata of RDS-related NRF

	**GA, weeks**					
	**25–27**	**28–31**	**32–33**	**34–36**	**37–38**	**39–42**
N	36	115	56	53	12	15
Male	22 (61.1)	66 (57.4)	33 (58.9)	30 (56.6)	9 (75.0)	13 (86.7)
BW, mean ± SD, g	1050 ± 179	1429 ± 296	1941 ± 351	2372 ± 557	2873 ± 522	3215 ± 763
Survival	17 (47.2)	83 (72.2)	50 (89.3)	50 (94.3)	11 (91.7)	13 (86.7)
Surfactant	29 (80.6)	87 (75.7)	35 (62.5)	29 (54.7)	7 (58.3)	8 (53.3)
Non-surfactant	7 (19.4)	28 (24.3)	21 (37.5)	24 (45.3)	5 (41.7)	7 (46.7)
Survival with surfactant	16 (55.2)	72 (82.8)	31 (88.6)	27 (93.1)	6 (85.7)	7 (87.5)
Survival without surfactant	1 (14.3)	11 (39.3)^***^	19 (90.5)	23 (95.8)	5 (100)	6 (85.7**)**
Difference, %^a^	40.9	43.5	−1.9	−2.7	−14.3	1.8
Numbers needed to treat^b^	2.4	2.3	–	–	–	56
	**BW, g**					
	**< 1000**	**1000–1499**	**1500–2499**	**2500–3999**	**≥4000**	**Total**
N	20	98	113	54	2	287
Male	6 (30.0)	58 (59.8)	64 (56.6)	44 (81.5)	1 (50.0)	174 (60.3)
GA, mean ± SD, weeks	27.6 ± 1.8	29.5 ± 2.1	32.5 ± 1.9	36.9 ± 2.2	37.1 ± 3.0	32.0 ± 3.5
Survival	6 (30.0)	64 (65.3)	103 (91.2)	49 (90.7)	2 (100)	224 (78.0)
Surfactant	16 (80.0)	75 (76.5)	73 (64.6)	31 (57.4)	0	195 (67.9)
Non-surfactant	4 (20.0)	23 (23.5)	40 (35.4)	23 (42.6)	2 (100)	92 (32.1)
Survival with surfactant	6 (37.5)	55 (73.3)	70 (95.9)	28 (90.3)	2 (100)	159 (81.5)
Survival without surfactant	0	9 (39.1)^**^	33 (82.5)^*^	21 (91.3)	2 (100)	65 (70.7)^*^
Difference, %^a^	37.5	34.2	13.4	−1.0	0	10.8
Numbers needed to treat^b^	2.9	2.9	7.5	–	–	9.3

All values are presented as mean ± standard deviation (SD) or n (%) referring to total number in each sub-group. Chi-square test was used for statistical analysis of differences in survival rate between surfactant- and non-surfactant-treated sub-groups

*Abbreviations*: *BW* Birthweight, *GA* Gestational age, *RDS* Respiratory distress syndrome, *NRF* Neonatal respiratory failure

*P < 0.05, *P < 0.01, ***P < 0.001 vs. survival with surfactant

^a^The attributable (absolute) risk of death rate (difference in percentile) between surfactant and non-surfactant treated sub-groups

^b^Estimated by reciprocal (reverse ratio) of attributable risk difference multiplied by 100 for surfactant benefit

### Perinatal-neonatal risks for death

The mortality odds evaluated by univariable logistic regression models (Table 4 and Table S2) showed that BW < 1000 g, pulmonary hemorrhage and PPHN were strongly (ORs > 6), while GA < 28 weeks, Apgar 5 min < 7, MAS, CA, NEC and MV were moderately (ORs 3–5), associated with mortality. In the multivariable models, BW < 1500 g, higher SNAPPE-II, MAS, CA, PPHN, NEC remained odds for death in NRF, with MAS and CA the highest (ORs > 30), followed by BW < 1500 g and NEC (ORs 7–8). The increment of SNAPPE-II (by every 10 point) was associated with a steady increase in mortality (OR = 2.29, *P* < 0.001). In contrast, BW ≥4000 g, born in level III hospitals, cesarean delivery, CPAP and MV were associated with the reduction of death odds.

**Table 4:** Uni- and multivariable logistic regression analysis of the perinatal-neonatal risks of NRF mortality

	Crude OR (95%CI)^**a**^	Adjusted OR (95%CI)^**b**^
BW < 1000 (g)	9.97 (3.68, 27.0)^***^	7.13 (1.70, 29.9)^**^
1000–1499	1.84 (1.16, 2.93)^*^	7.21 (2.83, 18.4)^***^
1500–2499	0.43 (0.26, 0.70)^**^	1.02 (0.45, 2.33)
2500–3999	1.00 (reference)	1.00 (reference)
≥ 4000	0.85 (0.28, 2.59)	0.13 (0.02, 0.98)*
Born in level III hospitals	0.53 (0.37, 0.77)^***^	0.44 (0.24, 0.81)^**^
Cesarean delivery	0.38 (0.26, 0.55)^***^	0.30 (0.17, 0.55)^***^
Higher SNAPPE-II	2.43 (2.09, 2.82)^***^	2.29 (1.89, 2.78)^***^
Respiratory distress syndrome	1.44 (1.00, 2.07)	3.56 (1.05, 12.1)^*^
Meconium aspiration syndrome	3.92 (1.30, 11.9)^*^	31.5 (4.81, 206)^***^
Pneumonia/sepsis (primary)	0.38 (0.24, 0.59)^***^	2.72 (0.80, 9.26)
Congenital anomalies	4.50 (2.78, 7.31)^***^	30.0 (7.55, 119)^***^
Pulmonary hemorrhage	11.9 (5.34, 26.7)^***^	3.42 (1.05, 11.1)^*^
PPHN	6.74 (3.76, 11.1)^***^	3.42 (1.05, 11.1)^*^
Patent ductus arteriosus	2.30 (1.37, 3.89)^**^	0.42 (0.16, 1.08)
Necrotizing enterocolitis	4.19 (2.72, 7.72)^***^	7.96 (3.28, 19.4)^***^
CPAP	0.17 (0.11, 0.25)^***^	0.09 (0.04, 0.20)^***^
MV	3.04 (2.08, 4.44)^***^	0.43 (0.22, 0.84)^*^

*Abbreviations*: *NRF* Neonatal respiratory failure, *OR* Odds ratio, *CI* Confidence interval, *BW* Birthweight, *SNAPPE-II* Score for neonatal acute physiology perinatal extension II, *PPHN* persistent pulmonary hypertension of the newborn, *CPAP* Continuous positive airway pressure, *MV* Mechanical ventilation

*P < 0.05, **P < 0.01 and ***P < 0.001 vs. reference

^a^Variables listed in the table are selected by backward stepwise logistic regression model, with the crude OR analyzed by univariable model

^b^Adjusted by all the variables listed in the table through multivariable logistic regression model (Hosmer-Lemeshow goodness of fit, *P* = 0.344)

## Discussion

The results of prevalence and outcome of NRF in Huai’an in 2015 depicted the efficiency of respiratory care of the regional tertiary centers and associated facilities in the context of perinatal-neonatal care paradigm for pregnant morbidities with high risk of exposure to their offspring. The survival of critically ill newborns, especially very and extremely low GA and BW infants remained a big challenge, despite surfactant and non-invasive ventilation showing benefits in patients with RDS. By logistic regression analysis, the improved survival of NRF was associated with born in the tertiary hospital, cesarean delivery and respiratory support. It should reflect an integrated strategy for the prenatal, peripartum and early postnatal care. RDS, MAS and CA became the leading odds for increased NRF mortality, which indicates a shift of clinical priorities for reducing high death risk population under local perinatal-neonatal network system in transition. The study design, protocol and definition of NRF were similar to the previous multicenter studies in nation-wide, provincial or trans-provincial perspectives (mainly based on the tertiary NICU admission), which facilitated temporal and spatial comparisons regarding the overall and specific care efficiency.

Based on the current study, we have shown a prevalence of 13.3‰ and mortality of 2.5‰ of NRF in the whole regional livebirths. Although the data were close to NRF in United States and Italy with prevalence of 18–22‰, and mortality of 2.0–3.2‰, in the mid-1990s, the CFR (18.4%) in our study was still higher than in those countries (11.1–14.6%), denoting development of the care system still lagged at least two decades behind [30, 31]. The high proportion of NRF deaths due to withhold/withdrawal from critical care compromised the overall outcome. As there was a trend of reduced stillbirths and increased livebirths from the studied population [6, 7], we anticipate that the proportion of the very preterm infants may continue to grow in the subsequent years. Therefore, the survival rate and quality in early infancy for the very and extremely preterm infants, should be the focus in future investigation [32–34]. It was corroborated that respiratory support played a key role in the neonatal critical care for better survival as seen in the early 1990s [33, 35, 36].

Compared to those livebirths with very preterm or very LBW, the BW in reference range still accounted for 30–40% NRF in proportion, and contributed to nearly 50% of total deaths, despite that the prevalence of NRF in term livebirths was significantly low (< 1%). For those term infants, the perinatal morbidities underlying NRF were associated with substantial proportion of congenital pneumonia and early onset sepsis as at-risk population from all the hospitalized. However, their occurrence as primary diagnosis in those NRF of very and extremely low GA or BW was low or none (Table 2, Table S1). As there underwent expansion for both facility and technique implementation, the diagnostic criteria and definition of common neonatal diseases at level II and III hospitals may not be consistent. There was also a concern that the overdiagnosis and associated overuse of antibiotics of potential infection at birth when information from maternal aspect was missing or not linked efficiently, especially encountered when NICU admissions were through inter-hospital transportation. For the major complications of NRF, acquired pneumonia/sepsis was complicated in 50–70% of extremely, and 30–40% very preterm/LBW NRF patients (Table 2, Table S1), whereas postnatal steroids were applied to < 15% of them. As there was a concern of postnatal steroids related high risk of late onset sepsis [37], the use of steroids seemed restricted in the critical care of the region.

The other major morbidities in term patients were MAS and CA, with CFR around 50% and adjusted ORs as high as 30 by multivariable logistic regression analysis. The declining trend of prevalence of MAS compared to previous NRF studies [16, 17, 19, 22, 23] indicated the progress in prevention of fetal distress and delivery resuscitation for severe asphyxia-associated meconium aspiration and prompt caesarean delivery for post-term or macrosomia. However, the highest CFR still highlighted the limitation of local neonatal critical care in management of PPHN, severe sepsis, or CA with cardiopulmonary failure.

By comparing the previous NRF studies (2004–2012) [16, 17, 19, 22, 23] (Table 5), current study showed a declined overall CFR, from 32.1% in 2004 to 18.4% in 2015 (current study), with more applications of surfactant (from 16.6% to 27.8%) and CPAP (from 52.6% to 80.8%), and higher proportion of female, very and extremely preterm/LBW infants. The total benefit of surfactant treatment in RDS estimated by the net difference in survival increment was similar to the previous ones (with overall NNT around 10). However, the benefit turned to be more prominent in those of BW < 1500 g (NNT 3.0). As for the primary underlying diseases of NRF, RDS remained the first leading cause (around 35–60%) and pneumonia/sepsis the second (20–35%), while the proportion of MAS declined (from 9.5% in 2004 to 1.6% in 2015) and CA increased (from 3.0% in 2004 to 10.2% in 2015). The occurrence of acquired infection and neurological impairment increased, as well as for BPD and NEC as preterm-associated complications.

**Table 5:** Comparison of prevalence, clinical management and outcome of NRF of current study with previous surveys

Year of original data	2004	2007	2008	2012	2010	2015
Network region	Nation-wide multicenter NICU	Hebei province multicenter NICU	Nation-wide multicenter NICU	Northwest multicenter NICU	Huai’an regional all in-hospital	Huai’an regional all in-hospital
NRF	1722	1875	6864	1324	556	788
Prevalence of NRF	13.2^a^	16.9^a^	19.7^a^	13.4^a^	6.8^b^	9.9^b^
Mortality rate	32.1	31.4	24.7	15.4	22.5	18.4
**Clinical characteristics**						
Male	75.5	72.5	70.9	65.7	71.6	60.4
GA, weeks (Range)	34.9 ± 4.1 (24–44)	35.0 ± 4.0 (24–43)	34.9 ± 3.9 (23–45)	35.6 ± 3.7 (23–44)	35.3 ± 3.6 (26–43)	34.5 ± 4.0 (25–42)
< 37 weeks	63.3	63.0	62.5	60.2	64.6	67.0
< 32 weeks	–	–	23.9	13.1	20.5	28.8
< 28 weeks	2.6	1.6	2.4	1.0	2.0	4.9
BW, g (range)	2309 ± 832 (650–6075)	2267 ± 804 (600–5500)	2314 ± 819 (600–6500)	2379 ± 727 (660–4810)	2433 ± 789 (800–4980)	2334 ± 886 (700–5700)
< 2500 g	59.7	60.9	57.9	59.0	65.3	57.5
< 1500 g	17.2	17.7	16.6	11.5	11.7	19.8
< 1000 g	1.9	2.0	2.3	1.0	1.0	2.5
SNAPPE-II	20 [8, 34]	20 [8, 35]	15 [6, 27]	7 [5, 18]	–	10 [5, 19]
**Respiratory therapy**						
Surfactant	16.6	28.7	26.8	15.9	14.0	27.8
CPAP	52.6	69.4	69.2	70.0	67.8	80.8
MV	61.2	46.7	57.9	32.4	46.9	44.2
Postnatal steroids	–	15.9	20.5	–	15.6	15.7
**Primary diagnosis**						
RDS	602 (35.0)	881 (47.0)	3010 (43.9)	488 (36.9)	333 (59.9)	287 (36.4)
Survival rate	66.2	67.3	76.2	78.6	70.9	78.4
Surfactant treatment	36.0	58.3	54.8	35.7	21.6	67.9
Survival rate						
Surfactant	78.8	73.7	79.9	84.2	–	81.5
Non-surfactant	63.5^**^	58.6^**^	71.8^**^	75.8^**^	–	70.7
Difference^c^	15.3	15.1	8.1	8.4	–	10.8
Numbers needed to treat	6.5	6.6	12.3	11.9		9.3
RDS with BW < 1500 g^d^	242 (40.2)	331 (37.6)	816 (27.1)	121 (24.8)	–	118 (41.1)
Survival rate	54.5	45.3	56.9	55.3	–	59.3
Surfactant treatment	43.4	43.2	64.2	49.5	–	77.1
Survival rate						
Surfactant	67.6	54.5	59.8	68.5	–	67.0
Non-surfactant	44.5^**^	42.7^**^	52.2^*^	53.6^**^	–	33.3^**^
Difference^c^	13.1	11.8	7.6	14.9	–	33.7
Numbers needed to treat	7.6	8.5	13.2	6.7	–	3.0
Pneumonia/sepsis	18.4	25.1	21.7	38.0	27.0	35.3
Meconium aspiration syndrome	9.5	7.8	7.0	2.2	1.4	1.6
Congenital anomalies	3.0	–	–	–	0.9	10.2
**Complications**						
Acquired pneumonia/sepsis	17.1	21.7	14.3	19.2	17.6	21.4
Neurologic impairment	3.5	2.8	3.9	12.5	1.1	19.9
Air leak	2.3	1.2	3.1	3.4	5.4	6.0
Bronchopulmonary dysplasia	1.5	0.4	1.8	1.3	0.2	9.1
Patent ductus arteriosus	–	1.7	5.1	3.1	–	9.5
Necrotizing enterocolitis	–	0.6	1.3	2.9	0.2	5.8
Retinopathy of prematurity	1.0	–	1.2	1.0	–	4.4
Reference	15	14	17	20	21	Current

All values are presented as mean ± standard deviation (SD), or median [interquartile range, IQR], or n (%) or rate (%) referring to total NRF number in each column unless otherwise indicated

*Abbreviations*: *NRF* Neonatal respiratory failure, *NICU* Neonatal intensive care unit, *GA* Gestational age, *BW* Birthweight, *SNAPPE-II* Score for neonatal acute physiology perinatal extension II, *CPAP* Continuous positive airway pressure, *MV* Mechanical ventilation, *RDS* Respiratory distress syndrome

*P < 0.05, **P < 0.01 vs. survival with surfactant

^a^Referring to all hospitalized neonates in the multicenter NICUs

^b^Referring to all hospitalized neonates in the whole region of Huai’an

^c^The absolute risk of death rate (difference in percentile) between surfactant and non-surfactant treated sub-groups

^d^The percentage in parentheses referring to all RDS in each survey

The region of Huai’an had been undergoing transition of the maternal-infant healthcare since 2010 with improvement in both care facility and the universal health insurance [6–12, 23], which was characterized by availability and affordability of advanced facility and medication, coverage of all critically ill newborn infants from birth with parental engagement including decision-making. These changes in association with altered benefit and risk factors impacted on the outcome of NRF, represented to a great extent the quality of practice of perinatal and neonatal care at medium to medium-high level of domestic regions [4, 5, 7, 9–12]. We therefore, speculate that some of the major findings may occur in subsequent years (2016–2025), in a great number of evolving regions with similar transition in SES and perinatal care. From geographic and socioeconomic point of view, we postulate that the overall results may be representative of, and relevant for, up to 20–25% of the population in the country, amounted to 250–350 million of domestic population [4]. Moreover, by assuming the perinatal-neonatal care of Huai’an in 2015 to be a paradigm of respiratory management, we attempt to estimate the prevalence of major morbidities and burden from all hospitalized patients and total livebirths. From an incidence of NRF in 13‰, 5‰ RDS, 10‰ requiring CPAP, 6‰ MV, and 4‰ surfactant, we deduce these figures corresponding to 195,000, 75,000, 150,000, 90,000 and 60,000, respectively, or more, in the contemporaneous whole country livebirths (15 million) [4]. Nevertheless, more studies are needed to validate the relevance for the > 300 nation-wide sub-provincial cities/regions with variable developmental stages in both maternal-infant healthcare and SES. This study indeed offered fundamental concept, methodology and datafile as strength.

The limitation, regarding the data reliability, may include under- or over-diagnosis and treatment, overt and occult, of major and minor respiratory morbidities. In the study design and data process, we managed to scrutinize clinical records in the retrospective data collection. Although we adjusted diagnosis according to the study protocol to balance the authenticity of original data with the diagnostic criteria and definitions, variations of the quality of practice among different facilities still existed. It revealed a real-world practice with respiratory support taking most of the major perinatal and neonatal morbidity and mortality into account. Even so, some risk factors were not controlled well from the data analysis due to sample size with stratification in the multivariable logistic regression models. Interpretation of the results should be cautious. Next, the mortality of NRF still had substantially high proportion of withhold/withdrawal of critical care though the health insurance was implemented. There was no socioeconomic and sociopsychological element engaged in the risk estimation. Further studies should include these factors into estimation for exposure risks and outcome in the perinatal-neonatal care in transition.

## Conclusion

The salient findings revealed efficiency of neonatal critical care in the management of NRF by associated risk estimates delineating respiratory support and surfactant use among all the hospitalized patients from regional livebirths. It should enable a comprehension of current neonatal critical care status at sub-provincial region, and may be extrapolated to a large proportion of domestic population taking SES and transitional perinatal-neonatal care into account. It requires further studies to validate from different regions for those requiring critical care with adequate respiratory support and management, especially very and extremely preterm births, for better survival.

## Supplementary Information

**Additional file 1.** The definitions of primary diagnosis as underlying disease of neonatal respiratory failure, and the criteria of care level of neonatal intensive care unit.

**Additional file 2: Table S1.** Primary diagnosis, intervention and outcome of NRF in GA strata.

**Additional file 3: Table S2.** The univariable logistic regression of other perinatal factors not included in the multivariable regression model.

## Data Availability

The datasets generated during and analyzed during the current study are not publicly available due to other concurrent studies based on the datasets but are available from the corresponding author on reasonable request.

## Abbreviations

BPD: Bronchopulmonary dysplasia
BW: Birthweight
CA: Congenital anomalies
CFR: Case fatality rate
CI: Confidence interval
CNY: Chinese Yuan
CPAP: Continuous positive airway pressure
GDP: Gross domestic production
HFOV: High frequency oscillatory ventilation
HMCHCC: Huai’an Maternal and Child Health Care Center
IQR: Interquartile range
IVH: Intraventricular hemorrhage
LBW: Low birthweight
LOHS: Length of hospital stay
LOV: Length of ventilation
MAS: Meconium aspiration syndrome
MV: Mechanical ventilation
NEC: Necrotizing enterocolitis
NICU: Neonatal intensive care unit
NNT: Numbers needed to treat
NRF: Neonatal hypoxemic respiratory failure
OR: Odds ratio
PND: Postnatal day
PPHN: Persistent pulmonary hypotension of the newborn
ROP: Retinopathy of prematurity
SD: Standard deviation
SNAPPE-II: Score for neonatal acute physiology perinatal extension II
TRIN: Temporary respiratory insufficiency of the newborn
