# Supplementary material for: Outcome of neonatal hypoxemic respiratory failure: a livebirth population-based retrospective survey
Source: BMC Pediatr. 2022 Sep 17;22:552. doi: 10.1186/s12887-022-03603-9 (PMC9482183; doi:10.1186/s12887-022-03603-9)
Supplement: Supplementary file 1 — Additional file 1. The definitions of primary diagnosis as underlying disease of neonatal respiratory failure, and the criteria of care level of neonatal intensive care unit. [file 12887_2022_3603_MOESM1_ESM.docx]

**Definitions of primary diagnosis as underlying diseases of neonatal respiratory failure (NRF)**

Respiratory distress syndrome (RDS) was for neonates with respiratory distress early after birth, persisted with deranged blood gas values and low oxygen saturation on pulse oximetry while oxygen and invasive and non-invasive ventilation are applied, and characteristic radiological findings denoting preterm lung related pathological image. Surfactant therapy often alter hypoxemic course. Meconium aspiration syndrome (MAS) referred to meconium staining of the amniotic fluid, meconium found from airway suction, face, mouth and noses, and compatible chest radiographic image. Congenital pneumonia referred to pulmonary infection acquired in utero or during birth, with early onset respiratory manifestation (within 3 postnatal days), radiological and laboratory evidence of infection. Those with maternal history of fever, antibiotic use, clinical and histological chorioamnionitis, long term premature rupture of membrane, and positive bacterial culture of maternal or fluid samples were high risk population. Community-acquired pneumonia referred to lung infection at home with onset beyond 3 postnatal days. Sepsis was classified as systemic infection with blood culture positive results, or negative culture but with all of the following three criteria fulfilled: (1) two or more infection-related clinical manifestations; (2) abnormal white blood cell count, C-reactive protein level, or procalcitonin level; and (3) antibiotics for ≥5 days. Temporary respiratory insufficiency of the newborn (TRIN) was associated with aspirated amniotic fluid, delayed fetal lung fluid clearance or transient tachypnea of newborn (TTN), respiratory depression caused by medications, post-asphyxial pulmonary edema, non-inducted C-section delivery, or a combination of etiologies that led to pulmonary maladaptation. Congenital anomalies were defined as major structural defects with lethality or high mortality or other serious medical or functional consequences. Primary diagnosis as severe intraventricular hemorrhage was based on cranial ultrasound with grades III and IV, on early postnatal life.

**Criteria of admission and care level of neonatal intensive care unit (NICU)**

Intensive care was for those after NICU admission with mild-to-moderate morbidities, including Apgar score <7 at 5 min of birth; birthweight (BW) >1000 g but <2100 g; hypoxemic condition requiring inspired fraction of oxygen (FiO_2_) <0.4, with or without non-invasive/assisted ventilation, or high flow device, with no time limit; NEC stage I or stable NEC requiring total parenteral nutrition; hyperbilirubinemia requiring phototherapy; mild hypoxemic-ischemic encephalopathy (HIE) not requiring hypothermic therapy; suspected or established pneumonia/sepsis requiring antibiotic use, fluid infusion; unstable oxygenation requiring long term oxygen therapy; patent ductus arteriosus (PDA) of hemodynamic significance in need of medication for closure; and in convalescence from intensive or critical care, etc.

Critical care was provided to those after NICU admission with moderate-to-severe perinatal and neonatal morbidities, such as Apgar score at 5 min <3, gestational age (GA) <28 week, BW <1000 g, IVH grade III-IV, RDS and pneumonia/sepsis requiring intratracheal/mandatory ventilation, hemodynamic instability requiring vasopressors or inotropes, or complicated with persistent pulmonary hypertension (PPHN) requiring inhaled nitric oxide; moderate to severe HIE requiring hypothermic therapy; hyperbilirubinemia requiring exchange transfusion, moderate-to-severe BPD, NEC stage II-III, neurological impairment (encephalopathy, periventricular leukomalacia) requiring robust monitoring and intervention; NEC, PDA and other pathologies requiring peri-surgical care, continuous renal replacement therapy, extracorporeal membrane oxygenation, etc.
