# Supplementary material for: Outcome of neonatal hypoxemic respiratory failure: a livebirth population-based retrospective survey
Source: BMC Pediatr. 2022 Sep 17;22:552. doi: 10.1186/s12887-022-03603-9 (PMC9482183; doi:10.1186/s12887-022-03603-9)
Supplement: Supplementary file 2 — Additional file 2: Table S1. Primary diagnosis, intervention and outcome of NRF in GA strata. [file 12887_2022_3603_MOESM2_ESM.docx]

**Table S1** Primary diagnosis, intervention and outcome of NRF in GA strata.

| **GA (weeks)** | **25-27** | **28-31** | **32-36** | **37-38** | **39-41** | **>42** | **Total** |
| --- | --- | --- | --- | --- | --- | --- | --- |
| Livebirth | 54 | 265 | 2195 | 12916 | 42998 | 628 | 59056 |
| Death at delivery^a^ | 12 (22.2) | 11 (4.2) | 4 (0.2) | 4 (0.0) | 4 (0.0) | 0 | 35 (0.1) |
| Hospitalization^a^ | 40 (74.1) | 241 (90.9) | 1660 (75.6) | 1900 (14.7) | 4076 (9.5) | 43 (6.8) | 7960 (13.5) |
| NRF cases^a^ | 39 (72.2) | 188 (70.9) | 301 (13.7) | 97 (0.8) | 158 (0.4) | 5 (1.2) | 788 (1.3) |
| SNAPPE-II | 21 [12, 26] | 7 [0, 18] | 5 [0, 14] | 12 [5, 21] | 21 [5, 26] | 17 [11, 55] | 10 [5, 19] |
| Non-survivor | 23 [15, 39] | 22 [12, 41] | 23 [10, 58] | 42 [17, 63] | 47 [27, 63] | - | 30 [16, 53] |
| Survivor | 19 [9, 23] | 5 [0, 14]^***^ | 5 [0, 12]^***^ | 7 [5, 16]^***^ | 5 [5, 16]^***^ | - | 5 [0, 14]^***^ |
| **Primary diagnosis** |  |  |  |  |  |  |  |
| Respiratory distress syndrome | 36 (92.3) | 115 (61.2) | 109 (36.2) | 12 (12.4) | 14 (8.9) | 1 (20.0) | 287 (36.4) |
| Survival^b^ | 17 (47.2) | 83 (72.2) | 100 (91.7) | 11 (91.7) | 12 (85.7) | 1 (100) | 224 (78.0) |
| Meconium aspiration syndrome | 0 | 0 | 2 (0.7) | 4 (4.1) | 6 (3.8) | 1 (20.0) | 13 (1.6) |
| Survival^b^ | 0 | 0 | 1 (50.0) | 2 (50.0) | 3 (50.0) | 1 (100) | 7 (53.8) |
| Pneumonia/sepsis | 2 (5.1) | 46 (24.5) | 104 (34.6) | 50 (51.5) | 75 (47.5) | 1 (20.0) | 278 (35.3) |
| Survival^b^ | 1 (50.0) | 40 (87.0) | 98 (94.2) | 46 (92.0) | 64 (85.3) | 1 (100) | 250 (89.9) |
| TRIN | 0 | 16 (8.5) | 56 (18.6) | 12 (12.4) | 23 (14.6) | 0 | 107 (13.6) |
| Survival^b^ | 0 | 15 (93.8) | 53 (94.6) | 9 (75.0) | 19 (82.6) | 0 | 96 (89.7) |
| Congenital anomalies | 1 (2.6) | 5 (2.7) | 21 (7.0) | 17 (17.5) | 34 (21.5) | 2 (40.0) | 80 (10.2) |
| Survival^b^ | 0 | 5 (100) | 17 (81.0) | 9 (52.9) | 13 (38.2) | 0 | 44 (55.0) |
| Intraventricular hemorrhage (III-IV) | 0 | 6 (3.2) | 9 (3.0) | 2 (2.1) | 6 (3.8) | 0 | 23 (2.9) |
| Survival^b^ | 0 | 6 (100) | 9 (100) | 2 (100) | 5 (83.3) | 0 | 22 (95.7) |
| **Respiratory interventions** |  |  |  |  |  |  |  |
| Surfactant | 30 (76.9) | 99 (52.7) | 72 (23.9) | 8 (8.2) | 10 (6.3) | 0 | 219 (27.8) |
| CPAP | 31 (79.5) | 175 (93.1) | 274 (91.0) | 67 (69.1) | 87 (55.1) | 3 (60.0) | 637 (80.8) |
| CPAP only | 10 (25.6) | 102 (54.3) | 217 (72.1) | 39 (40.2) | 60 (38) | 2 (40.0) | 430 (54.6) |
| CPAP & MV | 21 (53.8) | 73 (38.8) | 57 (18.9) | 28 (28.9) | 27 (17.1) | 1 (20.0) | 207 (26.3) |
| MV | 27 (69.2) | 83 (44.1) | 82 (27.2) | 58 (59.8) | 95 (60.1) | 3 (60.0) | 348 (44.2) |
| MV initial | 16 (41.0) | 28 (14.9) | 41 (13.6) | 35 (36.1) | 62 (39.2) | 1 (20.0) | 183 (23.2) |
| HFOV | 8 (20.5) | 17 (9.0) | 15 (5.0) | 19 (19.6) | 26 (16.5) | 0 | 85 (10.8) |
| Postnatal steroids | 9 (23.1) | 21 (11.2) | 27 (9.0) | 21 (21.6) | 43 (27.2) | 3 (60.0) | 124 (15.7) |
| **Major complications** |  |  |  |  |  |  |  |
| Acquired pneumonia/sepsis | 21 (53.8) | 60 (31.9) | 49 (16.3) | 13 (13.4) | 23 (14.6) | 3 (60.0) | 169 (21.4) |
| Neurological impairment | 15 (38.5) | 32 (17.0) | 62 (20.6) | 20 (20.6) | 27 (17.1) | 1 (20.0) | 157 (19.9) |
| Air leak | 0 | 2 (1.1) | 10 (3.3) | 16 (16.5) | 19 (12.0) | 0 | 47 (6.0) |
| Bronchopulmonary dysplasia | 17 (43.6) | 40 (21.3) | 10 (3.3) | 3 (3.1) | 2 (1.3) | 0 | 72 (9.1) |
| Pulmonary hemorrhage | 5 (12.8) | 1 (0.5) | 6 (2.0) | 8 (8.2) | 10 (6.3) | 0 | 30 (3.8) |
| Persistent pulmonary hypertension | 3 (7.7) | 4 (2.1) | 11 (3.7) | 14 (14.4) | 28 (17.7) | 1 (20.0) | 61 (7.7) |
| Patent ductus arteriosus | 10 (25.6) | 18 (9.6) | 14 (4.7) | 11 (11.3) | 21 (13.3) | 1 (20.0) | 75 (9.5) |
| Necrotizing enterocolitis | 6 (15.4) | 25 (13.3) | 11 (3.7) | 2 (2.1) | 2 (1.3) | 0 | 46 (5.8) |
| Retinopathy of prematurity | 7 (17.9) | 20 (10.6) | 6 (2.0) | 1 (1.0) | 1 (0.6) | 0 | 35 (4.4) |
| **Outcomes** |  |  |  |  |  |  |  |
| Length of ventilation, hour | 232 [62, 758] | 109 [50, 192] | 64 [41, 107] | 69 [34, 117] | 48 [30, 88] | 240 [41, 259] | 70 [39, 126] |
| Non-survivor | 89 [40, 389] | 31 [24, 124] | 46 [6, 103] | 46 [12, 108] | 61 [37, 90] | - | 41 [13, 117] |
| Survivor | 696 [222, 1020]^***^ | 114 [74, 195]^***^ | 64 [41, 107] | 71 [41, 120] | 22 [8, 70]^***^ | - | 72 [43, 134]^***^ |
| Length of hospital stay, day | 35 [6, 59] | 34 [19, 46] | 16 [13, 23] | 13 [6, 17] | 12 [5, 17] | 17 [4, 18] | 17 [11, 27] |
| Non-survivor | 11 [2, 30] | 3 [1, 10] | 3 [1, 5] | 3 [1, 6] | 2 [1, 4] | - | 3 [1, 8] |
| Survivor | 59 [42, 67]^***^ | 38 [27, 50]^***^ | 17 [14, 23]^***^ | 15 [10, 21]^***^ | 15 [11, 19]^***^ | - | 19 [14, 30]^***^ |
| Costs of stay, CNY, x10^3^ | 48 [12, 76] | 36 [20, 54] | 18 [13, 27] | 15 [8.8, 23] | 14 [8.6, 20] | 18 [6.0, 26] | 19 [12, 34] |
| Non-survivor | 18 [55, 49] | 9.8 [3.2, 18] | 6.8 [3.0, 12] | 5.8 [2.0, 14] | 6.1 [3.0, 11.5] | - | 6.8 [3.3, 15] |
| Survivor | 77 [50, 93]^***^ | 42 [30, 57]^***^ | 19 [14, 27]^***^ | 16 [10, 29]^***^ | 16 [12, 22]^***^ | - | 21 [14, 36]^***^ |
| Mortality | 21 (53.8) | 39 (20.7) | 23 (7.6) | 18 (18.6) | 42 (26.6) | 2 (40.0) | 145 (18.4) |
| 0-6 (PND) | 9 (23.1) | 24 (12.8) | 17 (5.6) | 13 (13.4) | 32 (20.3) | 0 | 95 (12.1) |
| 7-27 | 6 (15.4) | 13 (6.9) | 6 (2.0) | 4 (4.1) | 9 (5.7) | 2 (40.0) | 40 (5.1) |
| >28 | 6 (15.4) | 2 (1.1) | 0 | 1 (1.0) | 1 (0.6) | 0 | 10 (1.3) |
| Withdrawal of treatment | 12 (30.8) | 24 (12.8) | 15 (5.0) | 10 (10.3) | 19 (12.0) | 1 (20.0) | 81 (10.3) |

Abbreviations: NRF, neonatal respiratory failure; GA, gestational age; SNAPPE-II, score for neonatal acute physiology perinatal extension II; TRIN, transient respiratory insufficiency of the newborn; CPAP, continuous positive airway pressure; MV, mechanical ventilation; HFOV, high frequency oscillatory ventilation; CNY, Chinese Yuan.

All values are presented as median [interquartile ranges] or n (%), referred to total number in each column. ***P <0.001 vs. non-survivor.

a. Numbers refer to the total livebirths in each column.

b. Survival rate (%) refers to the specific case number listed above.
