# Supplementary material for: Outcome of neonatal hypoxemic respiratory failure: a livebirth population-based retrospective survey
Source: BMC Pediatr. 2022 Sep 17;22:552. doi: 10.1186/s12887-022-03603-9 (PMC9482183; doi:10.1186/s12887-022-03603-9)
Supplement: Supplementary file 3 — Additional file 3: Table S2. The univariable logistic regression of other perinatal factors not included in the multivariable regression model. [file 12887_2022_3603_MOESM3_ESM.docx]

**Table S2** The univariable logistic regression of other perinatal factors not included in the multivariable regression model.

|  | **OR** | **95% CI** | | **P** |
| --- | --- | --- | --- | --- |
| Admitted in level III hospitals | 1.13 | (0.25, 5.21) | | 0.876 |
| GA, 25-27 (weeks) | 3.22 | (1.57, 6.63) | | 0.001 |
| 28-31 | 0.72 | (0.44, 1.19) | | 0.202 |
| 32-36 | 0.23 | (0.13, 0.40) | | <0.001 |
| 37-38 | 0.63 | (0.34, 1.17) | | 0.144 |
| 39-41 | 1.00 (ref) | |  | |
| >42 | 1.84 | (0.30, 11.4) | | 0.512 |
| SGA | 0.83 | (0.36, 1.90) | | 0.661 |
| Male | 0.65 | (0.45, 0.93) | | 0.018 |
| Hypertensive disorder of pregnancy | 0.76 | (0.44, 1.31) | | 0.321 |
| Gestational diabetes of mellitus | 1.16 | (0.47, 2.91) | | 0.746 |
| Anemia | 0.64 | (0.39, 1.05) | | 0.077 |
| Premature rupture of membrane | 1.07 | (0.72, 1.59) | | 0.756 |
| Placenta abnormality | 0.81 | (0.45, 1.46) | | 0.480 |
| Umbilical cord abnormality | 1.49 | (0.89, 2.50) | | 0.133 |
| Antenatal steroids | 0.65 | (0.44, 0.96) | | 0.029 |
| Fetal distress | 0.85 | (0.32, 2.25) | | 0.739 |
| Amniotic fluid contamination | 1.96 | (1.25, 3.08) | | 0.003 |
| Apgar score 5 min<7 | 3.99 | (2.53, 6.30) | | <0.001 |
| Multiple births | 0.97 | (0.58, 1.64) | | 0.911 |
| DR resuscitation | 2.11 | (1.45, 3.08) | | <0.001 |
| Intensive care | 0.34 | (0.12, 0.97) | | 0.043 |
| Critical care | 2.36 | (0.92, 6.03) | | 0.073 |
| Admitted PND 1 | 0.55 | (0.33, 0.91) | | 0.019 |
| TRIN | 0.47 | (0.24, 0.90) | | 0.022 |
| Intraventricular hemorrhage (III-IV) | 0.20 | (0.03, 1.47) | | 0.112 |
| Acquired pneumonia/sepsis | 1.96 | (1.32, 2.93) | | 0.001 |
| Neurological impairment | 1.06 | (0.68, 1.66) | | 0.798 |
| Air leak | 1.76 | (0.91, 3.43) | | 0.095 |
| Bronchopulmonary dysplasia | 2.58 | (0.74, 8.92) | | 0.135 |
| Surfactant | 0.99 | (0.66, 1.48) | | 0.951 |
| Postanal steroids | 0.39 | (0.20, 0.74) | | 0.004 |

Abbreviations: NRF, neonatal respiratory failure; OR, odds ratio; CI, confidence interval; GA, gestational age; SGA, small for GA; DR, delivery room; PND, postnatal day(s); TRIN, transient respiratory insufficiency of the newborn.
